# Pulsatilla

**Published:** 1898-11

**Authors:** 


### HOMŒOPATHIC PHYSICIAN,

A MONTHLY JOURNAL OF

HOMŒOPATHIC MATERIA MEDICA AND CLINICAL MEDICINE.

If our school ever gives up the strict inductive method of Hahnemann, we are lost, and deserve only to be mentioned as a caricature in the history of medicine."—CONSTANTINE HERING.

Vol. XVIII. NOVEMBER, 1898. No. 11.

### EDITORIAL.

PULSATILLA.—The editorial in the October number was concluded with some notes on redness of the cheeks. The following additional indications are here given to complete that group of notes.

Arnica, Chamomilla, Ignatia, Natrum-muriaticum, all have redness of one cheek and paleness of the other.

Natrum-muriaticum, left cheek is red and the right one is pale.

Most Pulsatilla symptoms are on the right side.

Face may be bloated and purple under Pulsatilla. This is similar to Belladonna.

Under Puls. the lower lip is swelled and cracked in the middle. Calcarea-carb. has eruptions on lower lip.

Apis., Bell., and Calc-carb. have swelling of the upper lip.

Pulsatilla has looseness and painfulness of teeth. This is similar to Mercury.

Pulsatilla has sore gums. The pain is a *sore* pain. It is characteristic of Puls.

Pulsatilla has dryness of the mouth in the morning without thirst. This symptom is found under Bell., Lyc., Nux-mos., Sabadilla.

The characteristic tongue of Pulsatilla is coated yellow or white and slimy, or "covered with tough mucus."

The Belladonna tongue is dry and red.

Puls. has inflammation of throat with veins distended, similar to Kali-bichromicum.

Pulsatilla has desire for beer, but the beer tastes sweet, while butter tastes bitter.

Aversion to meat and fat food is characteristic of Pulsatilla. So, also is aversion to bread, and to milk.

The following notes may be found of value in connection with this symptom:

Aversion to rich, fat food, Angust., Bry., Carbo-a., Carbo-v., Colch., Cyclam., Hepar, Nat-mur., Petrol., Rheum., Sulphur.

Desire for fat food, Nux-vom., Nitric-acid.

Desire for ham fat, Mez.

Aversion to bread, Ars., Nat-c., Plumb., Puls., Stront.

Desire for bread, Ars., Nat-m., Plumb., Stront.

Desire for bread and butter, Ferrum and Mag-carb.

Aversion to butter, Ars., Carbo-v., Chin., Cycl., Meny., Petrol., Puls.

Desire for butter, Merc.

Aversion to milk, Æthus., Amm-carb., Bell., Bry., Calc-c., Carbo-v., Cina., Guaij., Ign., Nat-carb., Nux-v., Puls., Sulph., Tart-emet.

Aversion to mother's milk, Cina, Lach, Merc., Sil., Stann.

Pulsatilla is the most important remedy in the morning sickness of pregnancy.

Pulsatilla has pressure in the pit of the stomach after each meal, with vomiting of the ingesta.

Kali-bichromicum has nausea after a meal with heat all over the body, and giddiness excited by drinking and smoking, and relieved in the open air.

Pulsatilla is a remedy of great importance in peritonitis. Sulphur is also of great value in this disease.

Pulsatilla has colic and rumbling in upper abdomen and nausea.

Pulsatilla has painless rumbling of gas in the abdomen, Lycopodium has painful rumbling.

Pulsatilla has incontinence of urine, with involuntary discharge when coughing, passing wind, and during sleep.

Petroleum has constant dripping of urine.

Pulsatilla is an important remedy in gonorrhœa, when the discharge is greenish yellow.

Pulsatilla is a valuable remedy in dropsy of the scrotum.

Pulsatilla is a valuable remedy when the first menstruation is delayed. Kali-bichromicum, Baryta, and Sulphur are also indicated in this condition.

Pulsatilla is indicated in suppression of lochia. So also is Zinc.

Pulsatilla is indicated in suppression of the milk after child-birth.

Urtica-urens is also indicated when the milk does not appear after child-birth.

Pulsatilla has groaning or rattling breathing when asleep.

Pulsatilla has severe dry cough and so has Nux-vomica.

Pulsatilla has cough with expectoration during the day, but not at night. This is similar to Calcarea.

Pulsatilla has palpitation of heart with inability to endure bed covering. Must throw it off.

Pulsatilla has lumbago with groaning and grunting while lying in bed. Yet has severe pain if moved. The Pulsatilla patient wants to move about but can't on account of the pain. He screams with the pain when moved, yet his restlessness compels him to move a little bit so as to secure a new position. Dr. Lippe had a case of lumbago in which the symptoms occurred as here stated, and he made a brilliant cure with Pulsatilla. Yet other remedies must not be forgotten in these cases. Thus Rhus-toxicodendron has lumbago which is better for a little while in a certain position, but soon the pain becomes severe and he must move to a new position. Then he gets relief for a little while, but soon he must move again.

Natrum-sulphuricum has hip-pain, better in a certain position, but soon compels him to move again, the motion causing intense suffering.

Pulsatilla is an important remedy in chilblains.

Pulsatilla has hot inflammatory swelling of the knee.

This symptom recalls to the mind of the editor a case of severe inflammation of the knee joint, occurring in a tailor while standing before his cutting board. The pain came as suddenly as a stroke of lightning throwing him to the floor, where he groaned loudly with the agony. He was carried home and his physician, the writer of this article, was summoned. We found the joint extremely sensitive to touch, and highly swollen. The patient had the foot resting on a high chair. The limb could not be allowed to hang down or to rest on the floor, because the pain became intensified to an unbearable degree. This is a keynote for Pulsatilla. The pain was most violent on first beginning to move. But continued movement was attended with severe suffering. He would remain quiet for a while with a subsidence of the worst pain, when in spite of the intense aggravation from motion he would rise from his chair and attempt to cross the room. When asked why he did so, he said he was so restless that he must move notwithstanding the pain.

According to Hering, the Pulsatilla pains appear suddenly and disappear gradually. In this case the patient was stricken suddenly as if by lightning when at his work and apparently perfectly well.

We gave Pulsatilla 200 in water; the pain was relieved, and in two hours he was able to retire to his room. In two days he was able to go to work, and in two or three days more was perfectly well. This case was published in Dr. Skinner's journal, *The Organon*, Vol. III, No. 1, January, 1880, page 34, at the request of Dr. Lippe, who highly approved of it as an instructive instance of homœopathic prescribing.

